# COVID-19 vaccine knowledge, beliefs and attitudes among Oregon healthcare provider types

**DOI:** 10.1017/ash.2022.201

**Published:** 2022-05-16

**Authors:** Lisa Corley Stampke, Jessica Osborn, Judith Guzman-Cottrill

## Abstract

**Background:** During this pandemic, the public has struggled to navigate the abundance of COVID-19 vaccine misinformation, and it is unclear how this misinformation has affected medical providers and their recommendations for patients. We sought to understand differences in COVID-19 vaccine knowledge, beliefs, and attitudes among Oregon healthcare provider types and regions of practice (rural, suburban, urban). **Methods:** A 36-question survey was constructed using Qualtrics with consultation from a survey methodologist. The survey was reviewed and approved by OHSU IRB and distributed via listserv or social media posting to provider societies in Oregon, including nurse practitioners (NPs), naturopathic doctors (NDs), physician assistants (PAs), doctors of medicine (MDs), doctors of osteopathic medicine (DOs), or practioners with a bachelor of medicine–bachelor of surgery (MBBS), and via the Oregon Health Authority (OHA) immunization practice listserv. The survey accepted responses from July 9 to August 12, 2021. Participants were volunteers and responses were anonymous. **Results:** We collected 101 responses. Among them, 87 participants completed 100% of survey questions. Survey respondents were predominantly White females aged 41–50 years with an MD, DO, or MBBS. The overall COVID-19 vaccination rate of respondents was 94.6%. The vaccination rate was highest among the 4 NDs and 7 PAs at 100%, followed by 78 MDs, DOs, and MBBSs at 96.2%, and 12 NPs at 75%. Of NP respondents, 67% practiced rurally; 25.6% of MDs, DOs, and MBBSs practiced rurally; and 25% of NDs and 28.6% of PAs practiced rurally. In total, 22% of NPs did not feel comfortable recommending the COVID-19 vaccine to patients, compared to 1% of MDs, DOs, and MBBSs and 0% of NDs or PAs. All provider types had high rates of disagreement with the statement that the COVID-19 pandemic had increased their trust in vaccine safety: 44% of NPs; 29% of PAs; 25% of NDs; and 7% of MDs, DOs, and MBBSs. Among 19 rural providers, 19% indicated mistrust in public health to ensure that vaccines are safe versus 3% in suburban areas and 0% in urban areas. **Conclusions:** COVID-19 vaccine hesitancy is prevalent among healthcare providers and may be higher in NPs and those practicing rurally. Unfortunately, the response rate of NPs was low. Future research should focus on these providers to better understand their knowledge, beliefs, and attitudes about COVID-19 vaccines. These results can also inform future targeted vaccine education to healthcare providers during public health crises.

**Funding:** None

**Disclosures:** None

